# Evaluation of Implementing a Home-Based Fall Prevention Program among Community-Dwelling Older Adults

**DOI:** 10.3390/ijerph16061079

**Published:** 2019-03-26

**Authors:** Branko F. Olij, Vicki Erasmus, Lotte M. Barmentloo, Alex Burdorf, Dini Smilde, Yvonne Schoon, Nathalie van der Velde, Suzanne Polinder

**Affiliations:** 1 Department of Public Health, University Medical Center Rotterdam, Erasmus MC, 3000 CA Rotterdam, The Netherlands; v.erasmus@erasmusmc.nl (V.E.); l.barmentloo@erasmusmc.nl (L.M.B.); a.burdorf@erasmusmc.nl (A.B.); s.polinder@erasmusmc.nl (S.P.); 2 GENERO Foundation, 3001 AE Rotterdam, The Netherlands; dsmilde@xs4all.nl; 3 Department of Geriatric Medicine, Radboud University Medical Center, 6525 GC Nijmegen, The Netherlands; yvonne.schoon@radboudumc.nl; 4 Section of Geriatric Medicine, Department of Internal Medicine, Amsterdam, University of Amsterdam. UMC, Amsterdam Public Health Research Institute, 1105 AZ Amsterdam, The Netherlands; n.vandervelde@amc.uva.nl

**Keywords:** accidental falls, aged, prevention and control, exercise, independent living, implementation science

## Abstract

We aimed to describe and evaluate the implementation of a home-based exercise program among community-dwelling adults aged ≥65 years. In an observational study, the twelve-week program was implemented in a community setting. The implementation plan consisted of dialogues with healthcare professionals and older adults, development of an implementation protocol, recruitment of participants, program implementation, and implementation evaluation. The dialogues consisted of a Delphi survey among healthcare professionals, and of individual and group meetings among older adults. The implementation of the program was evaluated using the framework model RE-AIM. In the dialogues with healthcare professionals and older adults, it was found that negative consequences of a fall and positive effects of preventing a fall should be emphasized to older adults, in order to get them engaged in fall prevention activities. A total of 450 older adults enrolled in the study, of which 238 started the program. The process evaluation showed that the majority of older adults were recruited by a community nurse. Also, a good collaboration between the research team and the local primary healthcare providers was accomplished, which was important in the recruitment. Future fall prevention studies may use this information in order to translate an intervention in a research project into a community-based program.

## 1. Introduction

Fall-related injuries among older adults are recognized as a large burden to Western society [1], which is expected to increase, due to the ageing population. If the yearly increase of fall-related emergency department (ED) visits in the United States remains the same until 2030, the number of visits is expected to increase by 137%, to 5.7 million [2]. These increasing ED visits have a major impact on healthcare costs [3]. The impact of falls does not relate only to ED visits, but also to minor injuries treated at a general practitioner. Furthermore, falls have a large impact on the lives of older adults, as it can result in functional decline and loss of autonomy [4]. Many fall prevention programs have proven to be effective in reducing falls among older adults [5,6,7,8,9]. This mainly concerns multifactorial programs, which include an exercise component [5,6,7,8,9]. Furthermore, single exercise interventions have been proven to be effective in a community setting [7]. Effective group-based exercise programs are often offered on location, whereas older adults seem to favor individual-based programs offered in their homes [10]. This preference could be explained by the fact that group-based programs are generally more expensive, with less good accessibility [11]. Despite the existence of evidence-based programs, the successful implementation of fall prevention in the community remains a complex challenge [12]. There are only few studies that have evaluated the implementation of a fall prevention program. The majority of papers have focused on implementing fall prevention in a clinical setting [13,14,15,16]. Other studies have focused on the implementation of group-based exercise programs in the community [17,18,19]. To our knowledge, no study has evaluated the implementation of a home-based exercise program in the community. A review that evaluated five meta-analyses showed that effective implementation of a prevention program is strongly associated with better health-related outcomes [20]. Thus, evaluation of our implementation program could be helpful for effectively translating an intervention in a research project into successful community-based programs. The comprehensive evaluation framework model RE-AIM has been often used to evaluate implementation studies [21]. This model consists of five dimensions: reach, effectiveness, adoption, implementation, and maintenance. The aim of this paper was to describe and evaluate the implementation of a home-based exercise program in a community setting, among adults aged ≥65 years. Except for effectiveness, this paper describes the measures relating to the RE-AIM model.

## 2. Materials and Methods

### 2.1. Study Design and Population

**Figure 1:**
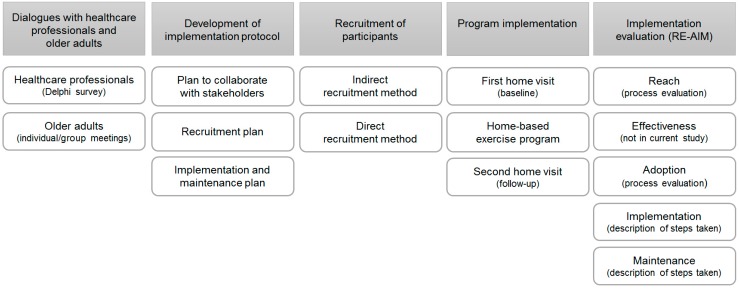
Steps taken to implement a home-based exercise program.

In the current observational study, a home-based exercise program was offered for twelve weeks to community-dwelling adults aged ≥65 years, living in the city of Breda, in the Netherlands. Older adults that did not understand the Dutch language, those with dementia, or those living in a residential care facility, were excluded. The implementation of the home-based exercise program consisted of multiple steps, which will subsequently be discussed (Figure 1).

Written informed consent was provided by all participants. The medical ethics committee of Erasmus MC, University Medical Center Rotterdam, waived ethical approval of the study (number 2017-139).

### 2.2. Dialogues with Heatlchare Professionals and Older Adults

Prior to the implementation phase, dialogues with healthcare professionals and older adults were performed. The aim of the dialogues was to develop strategies to implement a fall prevention program in a community setting, taking into account the local needs and infrastructure. These strategies were determined based on a Delphi survey that was performed among a panel of healthcare professionals, and based on individual and group meetings that were organized among a user panel of older adults. The measures of this twofold approach were used to develop the implementation protocol.

#### 2.2.1. Healthcare Professionals

A panel of healthcare professionals was consulted in a Delphi survey, consisting of two rounds of online questionnaires. This study was based on a previously published Delphi study [22]; however, questions were adjusted to the local settings of Breda and to the program that would be implemented. The Delphi panel consisted of community nurses, physiotherapists, occupational therapists, general practitioners (GPs), and geriatricians, from all over the Netherlands. The healthcare professionals were recruited through purposive expert sampling, in which the research groups’ personal network, the network of healthcare professionals, and websites on fall prevention were used. After the first questionnaire was completed by the panel, responses were summarised in order to develop a second questionnaire. This made it possible to elaborate on all topics. The questionnaires consisted of multiple choice and ranking questions. In the current manuscript, multiple choice data is reported as number and percentage, whereas data on ranking is reported as rank (i.e., ‘most’ (1) to ‘least’ (5 or 6)) and percentage. The level of consensus among the panel was considered as a frequency of ≥75% on multiple choice or ranking questions. The answers of healthcare professionals that completed the second round questionnaire were included in the data analysis, and are discussed in this manuscript. The online questionnaires were conducted using open-source LimeSurvey software [23]. Analyses were performed using SPSS Statistical Data software (IBM), version 24 (BO: International Business Machines Corporation (IBM), New York, United States).

#### 2.2.2. Older Adults

A user panel of community-dwelling adults aged ≥65 years, living in Breda, was consulted in individual and group meetings. The user panel was recruited by local community nurses. Initially, all members of the user panel were asked to participate in a group meeting. However, as some individuals had trouble walking, those individuals were visited at home, by a member of the research team. The group meetings took place in community centers, located near the homes of the older adults, and were led by a member of the research team. Another member of the research team was present to take notes during the group meetings. The questions of the individual and group meetings were drafted together with another panel of older adults, in a participatory design approach. This user panel consisted of older adults that were part of an ‘older adult forum’, which aims to improve the quality of life and care of older adults. The topics that were discussed during the individual and group meetings were about the barriers and facilitators for participating in fall prevention activities, and about the individuals considered key in fall prevention. The notes that were taken during the meetings by a member of the research team were used to identify and cluster specific themes. These themes were summarized, which are reported descriptively in the current paper.

### 2.3. Development of Implementation Protocol

The implementation protocol consisted of multiple topics such as a plan on the structural collaboration with stakeholders and a recruitment plan to determine which methods should be used to recruit the study population. Furthermore, a plan for practical implementation and maintenance of the program was included, which consisted of the steps taken to embed tasks concerning the implementation of the program in local organizations. During the study period, the protocol was revised and adjusted when necessary.

### 2.4. Recruitment of Participants

The recruitment of participants consisted of indirect and direct methods. Indirectly, older adults were recruited for the study by a press release, advertisements in local papers, and commercials broadcasted on a local television and radio channel. Word of mouth resulted in older adults being recruited, as well. Directly, older adults were recruited with a personal approach by primary healthcare providers, such as community nurses, physiotherapists, occupational therapists, and GPs. Also, information sessions and workshops for older adults were held by the research team in community centers, and flyers about the study were distributed at senior living apartments and in shopping malls. As reported previously, older adults with dementia were excluded from participating. All primary healthcare providers that were involved in the recruitment of older adults were informed about this exclusion criteria. By informing these stakeholders, an attempt was made to minimize the chances of an older adult with dementia enrolling. In the current paper, a distinction is made between older adults that ‘enrolled’ and ‘participated’ in the study. The number of older adults that ‘enrolled’ in the study corresponds to the number of applications that were received by the research team by telephone, regular mail, or email. The number of older adults that ‘participated’ in the study corresponds to the number of individuals that started the twelve-week home-based exercise program.

### 2.5. Program Implementation

All participants were offered a home-based exercise program, which was based on the Senior Step intervention [24]. In the Senior Step intervention, participants performed self-tests for assessing mobility and fall risk. The safety and feasibility of these tests were evaluated in that study. Apart from the self-tests, participants were also offered an instruction book with exercises. The instruction book was developed by two physiotherapists, and was based on the Otago program [25]. The book consists of exercises to: (1) promote safe use of walking aids, (2) improve mobility (e.g., standing up from a chair), (3) improve reaching (i.e., forwards, sideways, and backwards), (4) improve quality of walking and walking speed, and (5) improve overall fitness (i.e., agility, strength, balance, and conditioning). The book is divided into four levels, ranging from simple, low intensive to complex, intensive exercises. In the current study, the instruction book of the Senior Step intervention was offered to participants as a home-based exercise program, for twelve weeks. During a first home visit at baseline, a member of the research team offered and explained the instruction book to the participant. The participant was advised by the research team on the exercise ‘level’ to start with, based on their mobility. Though, during the study period, the participant could themselves change the type and duration of exercises. During the first home visit at baseline, an ‘assessing care of vulnerable elders’ (ACOVE) questionnaire was given to the participant. This questionnaire evaluated the provided healthcare after a fall, in the previous 12 months [26]. It was used to create a baseline situation of the local fall-related healthcare. After twelve weeks of follow-up, a second home visit with a member of the research team took place. During this home visit, the instruction book was returned to the research team and the exercise program was evaluated with the participant by a questionnaire.

### 2.6. Implementation Evaluation

In order to describe and evaluate the implementation of the home-based exercise program, the comprehensive framework model RE-AIM was used. This model consists of the dimensions of reach, effectiveness, adoption, implementation, and maintenance [21]. In general, by evaluating the different dimensions within a study, information regarding the translation of research to practice is gained [21]. The original RE-AIM dimension definitions and the definitions used in the current study are presented in Table 1. The dimension ‘reach’ was assessed through process evaluation, as the proportion of older adults enrolled in the study through indirect and direct methods. This information was gathered by asking every older adult that enrolled in the study how they were recruited. The barriers and facilitators in recruiting participants were also described for this dimension. The dimension ‘effectiveness’ is not discussed, as it was not the focus of the current paper. More information about the effectiveness of the program can be found in an earlier paper about the study. (Unpublished observations, by Branko F. Olij, Lotte M. Barmentloo, Dini Smilde, Nathalie van der Velde, Suzanne Polinder, Yvonne Schoon, and Vicki Erasmus.) The main findings of that paper were that 52% of the participants indicated that they frequently took part, which means that the exercises given in the instruction book were performed daily or a few days per week during the study period. Furthermore, the analyses indicated that a higher degree of pain was associated with frequent participation; however, frequent participation resulted in better health perceptions, over time. The activities that were executed to optimize collaboration between the research team and different local stakeholders were described within the dimension ‘adoption’. ‘Implementation’ was assessed through a process evaluation, as it was defined by the extent to which the twelve-week program was realized as planned. Also, program satisfaction of the participants was reported. As described in the previous paragraph, this information was based on a questionnaire that was administered after twelve weeks of follow-up. Questions included how much the participants liked the program; how useful they evaluated it to be; and whether the participants noticed a change in their risk awareness, confidence in balance, and in their level of physical activity during the study period. The number (n) and percentage (%) are reported for these measures, and the analyses were performed using SPSS Statistical Data software (IBM), version 24. Lastly, in order to evaluate the maintenance of the program, a description was given of the steps taken to embed the tasks concerning the implementation of the program in local organizations.

## 3. Results

The implementation plan components ‘dialogues with healthcare professionals and older adults’, ‘program implementation’, and ‘implementation evaluation’ will subsequently be discussed in the results section (Figure 1).

### 3.1. Dialogues with Healthcare Professionals and Older Adults

#### 3.1.1. Healthcare Professionals

A total of 129 healthcare professionals participated in the Delphi survey. The first questionnaire was completed by 81% (*n* = 105/129) of the panel, whereas the second questionnaires was completed by 74% (*n* = 95/129). According to the panel, the most important barrier in organizing fall prevention in a community setting is reaching older adults that are not in touch with healthcare professionals (*n* = 55/95; 58%) (Table 2). Poor communication between different stakeholders (*n* = 50/95; 53%) and absence of a coordinator (*n* = 49/95; 52%) were mentioned as barriers, as well. Important facilitators in organizing fall prevention in a community setting are good cooperation between different healthcare professionals (*n* = 57/95; 60%) and taking into account the wishes and needs of older adults (*n* = 57/95; 60%) (Table 2). The panel was also asked which individuals were considered key in organizing fall prevention in a community setting. A neighborhood care team (*n* = 26/95; 27%), the physiotherapist (*n* = 23/95; 24%), and the community nurse (*n* = 22/95; 23%) were most mentioned. A perceived barrier for participating in a fall prevention program was that older adults are not aware of the possibilities in their neighborhood (*n* = 32/93; 34% (rank 1)). Consensus was reached on facilitators to increase participation. Namely, emphasizing to older adults that fall prevention is important in maintaining functional independence was mentioned by 77% of the panel (*n* = 72/94; (rank 1)). Furthermore, an effective measure to increase participation rates among older adults is to raise awareness on the consequences of a fall (*n* = 43/94; 46% (rank 1)). Both the GP (*n* = 32/94; 34% (rank 1)) and the informal caregiver (*n* = 28/94; 30% (rank 1)) were considered key individuals in stimulating participation among older adults.

#### 3.1.2. Older Adults

In total, three individual and four group meetings with user panel members took place among a total of 27 older adults living in Breda. Three individual meetings took place in the homes of older adults, whereas four group meetings took place in community centers among 24 older adults. In every group meeting, four to eight older adults were present. Several barriers of participating in fall prevention activities were identified by the older adults during these meetings. Chronic pain, fear of participating, program costs, poor accessibility, and unawareness of their own fall risk were most often mentioned. Increasing awareness of the personal relevance of fall prevention was identified as an important facilitator. The user panel mentioned that their awareness increased after they had fallen. In order to raise awareness and increase participation rates among other older adults, the panel suggested emphasis on the negative consequences of a fall. Also, the positive effects of preventing a fall, such as maintaining functional independence, should be stressed upon. Furthermore, according to the user panel members, it was considered important to offer advice (and not frighten) to older adults concerning fall prevention activities. This way, they can make a deliberate choice without feeling forced to do so. Another strategy considered effective in engaging older adults in fall prevention activities is by involving a trusted individual, such as the community nurse, informal caregiver, or neighbor. Such an individual was thought to be different for every older adult, but was perceived to be of help in stimulating participation.

### 3.2. Program Implementation

A total of 450 older adults enrolled in the study, of which 238 older adults started the twelve-week home-based exercise program. Additional information on the baseline characteristics of older adults that enrolled the study, but did not participate, is provided in Supplementary Materials Table S1. At baseline, 52% (*n* = 124/238) of the participants were reported to have had a fall in the past twelve months. Out of these participants, 59 participants reported that they had visited a GP or an Emergency Department following the fall. In 12% of these 59 participants, an eye exam was performed, 15% was given a memory test, 25% was given a balance test, 22% was given advice about safety in and around the house, and 22% of the participants was advised by their physicians to start an exercise program. These results show that a limited number of participants were offered specific care to prevent a subsequent fall, prior to the implementation of the exercise program in their community.

### 3.3. Implementation Evaluation

#### 3.3.1. Reach

##### Indirect and Direct Methods

The indirect recruitment methods potentially reached 122,000 older adults. The majority of these older adults (*n* = 70,000) were reached through press releases about the study. Directly, over 3100 older adults were potentially reached. Recruitment through a community nurse (*n* = 1220) and through flyers about the study (*n* = 1000) resulted in the majority of these older adults being reached. As reported in the Methods section, every older adult that enrolled in the study was asked how they were recruited. A total of 450 older adults enrolled in the study, of which 290 indicated the method of their recruitment. Twelve older adults indicated to have been recruited through an indirect method and 278 older adults through a direct method. Of the individuals recruited through a direct method, the majority (*n* = 233) were recruited through a community nurse.

##### Barriers and Facilitators

Firstly, a barrier in recruiting participants was that, although several older adults were contacted, relatively few enrolled in the study. Indirect methods, particularly, resulted in a small number of older adults being enrolled in the study. Secondly, even though many local GPs were asked to be involved in the recruitment of study participants, not many were active in doing so. An exception being that by contacting practice nurses with specialization in care for older adults, it was possible to involve a few local GPs in the recruitment of study participants. Third, collaboration with the local government was difficult. Reasons for the local government not being involved in the study was because fall prevention was not high on their agenda, and there also was a lack of budget.

One recruitment method was identified as a facilitator in recruiting participants—personal approach by a community nurse encouraged older adults to engage in fall prevention activities, which resulted in the majority of enrolments. Another identified facilitator was the huge support for implementation of the program by local, primary healthcare providers. Optimizing a collaboration between the research team and these healthcare professionals was important in reaching and recruiting older adults.

#### 3.3.2. Adoption

Several activities were executed to optimize collaboration between the research team and local stakeholders, and facilitate adoption of the program. At the start of the study, neighborhood profiles were developed, which consisted of information on age distribution and socio-economic status of the inhabitants, and of a list of stakeholders we could potentially collaborate with. Then, a network with relevant stakeholders in every neighborhood was set up. This network consisted of, amongst others, local primary healthcare providers (i.e., community nurses, physiotherapists, occupational therapists, GPs, and practice nurses), older adult unions, local initiatives for older adults, and volunteers. The network of stakeholders acted as an advisory body. Namely, during the study period, meetings were organized in order to discuss several topics related to the local implementation of the exercise program. This was particularly related to the recruitment of older adults. Also, the wishes, needs, and expectations of all stakeholders were discussed. In order to keep all stakeholders informed, a quarterly newsletter was sent to them and others interested.

#### 3.3.3. Implementation

##### Implemented as Planned

The twelve-week home-based fall prevention program was, except for two elements, implemented as planned. Two necessary adaptations were made during the program. Originally, adults were recruited in two Breda neighborhoods. As it became clear that relatively few older adults would enrol in the study, recruitment was extended to a total of eight neighborhoods. Furthermore, eight participants did not agree to a second home visit after twelve weeks. These participants received a follow-up questionnaire by regular mail instead.

##### Program Satisfaction

**Figure 2:**
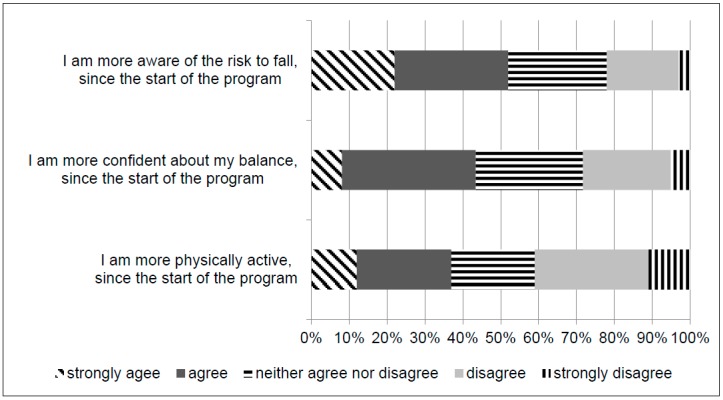
Program satisfaction of the participants after the twelve-week home-based exercise program.

A questionnaire on program satisfaction was conducted among the participants that completed the twelve-week program. Fifty-nine percent of the participants said that they moderately or strongly liked the program. The program was evaluated as at least moderately useful by 71% of the participants. Fifty-two percent of the participants agreed or strongly agreed with noticing a change in the awareness of their fall risk during the study period (Figure 2). Forty-three percent agreed or strongly agreed with noticing an increased confidence in their balance, and 37% agreed or strongly agreed with noticing a change in their level of physical activity during the study period.

#### 3.3.4. Maintenance

In order to maintain the home-based exercise program locally, an invitational conference was organized after the study period. This conference included all relevant stakeholders, such as older adults living in Breda, the municipality, a health insurer, home care organizations, local initiatives, GP representatives, a hospital, and physical and occupational therapists. During the conference, a presentation was made by the research team on the process and results of implementing the exercise program in the community. A discussion followed, which led to three stakeholders with interest in taking over the responsibilities of structurally implementing and financing the fall prevention program locally.

## 4. Discussion

The current paper describes and evaluates the implementation of a home-based exercise program among older adults. The dialogues with healthcare professionals and older adults showed that, in order to stimulate participation rates among older adults, the negative consequences of a fall should be emphasized. The positive effects of preventing a fall, such as maintaining functional independence, should be emphasized, as well. According to healthcare professionals and older adults, the informal caregiver is a key individual in stimulating participation among older adults. Using the framework model RE-AIM, the evaluation of implementing the home-based exercise program showed that many older adults were potentially reached, but relatively few enrolled in the study. Personal recruitment by their own community nurse was the most effective method of recruiting older adults. Furthermore, in the recruitment of older adults, a good collaboration between the research team and the local primary healthcare providers was important.

As reported previously, the current home-based exercise program was based on the intervention ‘Senior Step’. In the evaluation study of this intervention, an information meeting was much more effective in reaching and recruiting participants than a personal approach [27]. This is in contrast to our study, as we did recruit some participants by providing information sessions, but the majority of participants were recruited face-to-face by a community nurse. An explanation for this difference could be that, in the ‘Senior Step’ study, older adults living in a residential care facility were recruited as well. A study on an integrated neighborhood approach to support community-dwelling older adults in the Netherlands showed that community engagement is very important in the adoption of a program [28]. Likewise, a study on the implementation of a fall prevention program in the United States reported that having solid community partnerships is essential for good program adoption by stakeholders [17]. These results correspond to our study, as creating broad support among local stakeholders proved to be important in successfully implementing the program. Keeping stakeholders informed during the study period has been found to be important in the implementation success of a previous fall prevention program, as well [13]. By sending newsletters and by organizing network meetings in our study, we tried to inform stakeholders, as well. The network meetings were important to take the wishes and needs of the local stakeholders of different neighborhoods into account, in order to properly adopt the program. This relates to a study by Peel et al. (2017), who reported that neighborhoods differ in many characteristics, so it is important to take into account the needs of the local area, in order to effectively adopt and implement a program [15].

The healthcare professionals of the Delphi survey considered the GP as a key individual in stimulating participation among older adults. However, involving GPs in the recruitment of participants proved to be difficult in our study. A study by Brach et al. (2013) on a home-based exercise program for older adults reported that they recruited GPs by contacting a research network [29]. The involvement of GPs in the recruitment of older adults proved to be beneficial in their study. Perhaps, if we had contacted GPs through a research network, a better collaboration could have been established. Apart from the recruitment of older adults, GPs could also have an important role in the adoption of an intervention. Previous research in the Netherlands has shown that the GP can have a central role in multidisciplinary teams, focused on the care for older adults [30]. The paper identifies several competences that are key for a successful GP role, such as leadership and networking. A GP role with those competences could be of added value to the adoption of a fall prevention intervention.

After twelve weeks of follow-up, relatively low satisfaction of the program was reported by the participants, as 59% said that they liked the program. We have not asked why an individual did or did not like the program; however, a possible explanation for the relatively low satisfaction might be that older adults were missing support during the study period. Even though a member of the research team visited the participant twice at home (i.e., at baseline and after twelve weeks), no contact was made with the participant during the study period. Previous research has shown that support from others is considered important in the promotion and adherence of fall prevention interventions [12].

A strength of our study is that the comprehensive evaluation framework RE-AIM was used in order to evaluate the implementation. An evaluation such as this is valuable in translating research into practice. Also, using the different dimensions makes issues related to implementation more explicit, which is sometimes ignored in a more traditional presentation of results. A limitation of the current study is that processing qualitative data of the individual and group meetings with older adults could have been improved. Even though specific themes that emerged from the meetings were identified and clustered, no rigorous content analysis was performed, as it stretched beyond the scope of this study. This analysis could have yielded much richer insights of the important factors for fall prevention and should certainly be included in future research. Even though many older adults were potentially reached, relatively few enrolled in the study. This low willingness to participate could have resulted in selection bias. Nevertheless, despite not many older adults enrolling in the study, an increase in the awareness of fall prevention could still have been realized among this group. As Russell et al. (2017) have shown, the presence of risk awareness does not necessarily lead to older adults being willing to participate in fall prevention [31]. Another limitation of the study is that no information was available on what type or duration of exercises of the instruction book were performed by the participants. Thus, we were unable to determine what type or duration of exercises may have contributed to the effectiveness of the program. Participants of the current study might not be representative of a general population of community-dwelling older adults. Specifically, as the majority of older adults were recruited through healthcare professionals, the participants of the current study might be older and more frail than the general older adult population. Furthermore, relatively few participants with a migration background were included in the study. Only 6% of the older adult population of Breda has a migration background [32], but in other Dutch cities, this percentage is generally higher. The fact that individuals not understanding the Dutch language were excluded made it difficult for older adults with a migration background to enroll in our study. Even though ethnic minorities are often underrepresented in health research [33], some studies have shown effective methods to recruit immigrant participants. Namely, by providing bilingual program information, and by facilitating partnerships with community organizations, recruitment of immigrant participants could be improved [34,35]. Future implementation studies should take into account these methods in the planning of their interventions.

## 5. Conclusions

This study shows that the negative consequences of a fall and the positive effects of preventing a fall should be emphasized to older adults, in order to get them engaged in fall prevention activities. Furthermore, the importance of a good collaboration between the research team and local primary healthcare providers has been identified. Also, particularly community nurses can successfully help in reaching and recruiting older adults for a fall prevention program. All lessons learned in the current study could help and guide future interventions of research projects to successfully translate into community-based programs. As the population ages and absolute numbers of fall-related injuries keep increasing, implementing successful prevention programs will become more and more important in reducing healthcare costs.

## Figures and Tables

**Table 1:** Original definitions of RE-AIM dimensions: reach, adoption, implementation, and maintenance [21], and definitions of current study.

Dimension	Original Definition	Study Definition
Reach	Proportion of individuals that participated in the program	Proportion of individuals that enrolled in the study through indirect and direct methods; barriers and facilitators in recruitment
Effectiveness	Outcome effects of implementing the program as planned	Not discussed in current manuscript
Adoption	Proportion of practices and individuals that adopted the program	Activities executed to optimize collaboration with stakeholders
Implementation	Extent to which the program is implemented as planned	Extent to which the program was implemented as planned; program satisfaction of the participants
Maintenance	Extent to which a program is maintained over time	Activities executed to maintain the program locally

**Table 2:** Barriers and facilitators according to healthcare professionals in organizing fall prevention in a community setting.

	(*n* = 95)
Barrier	(%)
Reaching older adults that are not in touch with healthcare professionals	58
Poor communication between different stakeholders	53
Absence of a neighborhood coordinator	52
Healthcare professionals that do not have enough knowledge on fall prevention	47
High costs	36
Lack of time	31
Lack of a central location in a large neighborhood	15
Facilitator	(%)
Good cooperation between different healthcare professionals	60
Taking into account the wishes and needs of older adults	60
Clear communication between different stakeholders	48
Shared vision on fall prevention in a community setting among stakeholders	42
Word of mouth	31
Providing good information about fall prevention to healthcare professionals	28
Neighborhood coordinator that takes control	21

## Supplementary Materials

The following are available online at https://www.mdpi.com/1660-4601/16/6/1079/s1, Table S1: Baseline characteristics of older adults that applied for the study, but that did not participate.
