# Supplementary material for: Evaluation of Implementing a Home-Based Fall Prevention Program among Community-Dwelling Older Adults
Source: Int J Environ Res Public Health. 2019 Mar 26;16(6):1079. doi: 10.3390/ijerph16061079 (PMC6466172; doi:10.3390/ijerph16061079)
Supplement: Supplementary file 1 [file ijerph-16-01079-s001.pdf]

Supplementary Materials

Table S1. Baseline characteristics of older adults that applied for the study, but that did not participate.

	Total (n = 212)	No Informed Consent (n = 180)	Dropped Out before Start (n = 32)
	n (%)	*n (%)*	*n (%)*
Female	136 (65) [a]	116 (65) [e]	20 (65) [g]
Age-mean ± SD	82.3 ± 8.5 [b]	82.2 ± 8.6 [f]	82.7 ± 7.6 [g]
Living alone	144 (69) [c]	123 (69) [e]	21 (66)
Elevated fall risk [1]	26 (81)	n.a. *	26 (81)

SD: Standard deviation; [1] The fall risk test (part of a fall analysis assessment) determined that an elevated fall risk was present when a participant had a fall in the past twelve months, or the participant had mobility problems and a fear of falling [1,2]; [a]: n = 209; [b]: n = 195; [c]: n=210; [d]: n = 32; [e]: n = 178; [f]: n =164; [g]: n = 31.

References

1. Veiligheid, N.L. Valanalyse Inventarisatie Valrisico 65+ Door de Eerstelijnszorg [Fall Analysis Assessment Fall Risk 65+ by Primary Care]. 2018. Available from: https://www.veiligheid.nl/valpreventie/interventies/screening/valanalyse (accessed on 15 December 2018). (In Dutch)
2. Richtlijnen Database. *Preventie van valincidenten bij ouderen [Falls Prevention among Older Adults]*; 2017. (In Dutch)
